# Clinical indications for image-guided interventional procedures in the musculoskeletal system: a Delphi-based consensus paper from the European Society of Musculoskeletal Radiology (ESSR)—part VII, nerves of the lower limb

**DOI:** 10.1007/s00330-021-08283-0

**Published:** 2021-09-28

**Authors:** Luca Maria Sconfienza, Miraude Adriaensen, Domenico Albano, Andrea Alcala-Galiano, Georgina Allen, Maria Pilar Aparisi Gómez, Giacomo Aringhieri, Alberto Bazzocchi, Ian Beggs, Vito Chianca, Angelo Corazza, Danoob Dalili, Miriam De Dea, Jose Luis del Cura, Francesco Di Pietto, Elena Drakonaki, Fernando Facal de Castro, Dimitrios Filippiadis, Salvatore Gitto, Andrew J. Grainger, Simon Greenwood, Harun Gupta, Slavcho Ivanoski, Monica Khanna, Andrea Klauser, Ramy Mansour, Silvia Martin, Vasco Mascarenhas, Giovanni Mauri, Catherine McCarthy, David McKean, Eugene McNally, Kalliopi Melaki, Carmelo Messina, Rebeca Miron Mombiela, Ricardo Moutinho, Cyprian Olchowy, Davide Orlandi, Raquel Prada González, Mahesh Prakash, Magdalena Posadzy, Saulius Rutkauskas, Žiga Snoj, Alberto Stefano Tagliafico, Alexander Talaska, Xavier Tomas, Violeta Vasilevska Nikodinovska, Jelena Vucetic, David Wilson, Federico Zaottini, Marcello Zappia, Amanda Isaac

**Affiliations:** 1 IRCCS Istituto Ortopedico Galeazzi, Milan, Italy; 2 Dipartimento Di Scienze Biomediche Per La Salute, Università Degli Studi Di Milano, Via Riccardo Galeazzi 4, 20161 Milan, Italy; 3 Department of Medical Imaging, Zuyderland Medical Center, Sittard-Geleen, Heerlen, Brunssum, Kerkrade, the Netherlands; 4 Sezione Di Scienze Radiologiche, Dipartimento Di Biomedicina, Neuroscienze E Diagnostica Avanzata, Università Degli Studi Di Palermo, Palermo, Italy; 5 Hospital Universitario, 12 de Octubre, Madrid, Spain; 6 St Luke’s Radiology Oxford Ltd, Oxford, UK; 7 University of Oxford, Oxford, UK; 8 Department of Radiology, Auckland City Hospital, Auckland, New Zealand; 9 Department of Radiology, Hospital Vithas Nueve de Octubre, Valencia, Spain; 10 Diagnostic and Interventional Radiology, Department of Translational Research and New Technologies in Medicine and Surgery, University of Pisa, Pisa, Italy; 11 Diagnostic and Interventional Radiology, IRCCS Istituto Ortopedico Rizzoli, Bologna, Italy; 12 Analytic Imaging, Edinburgh, UK; 13 Ospedale Evangelico Betania, Napoli, Italy; 14 Clinica Di Radiologia EOC IIMSI, Lugano, Switzerland; 15 South West London Elective Orthopaedic Centre (SWLEOC), Epsom & St Helier University Hospitals NHS Trust, London, UK; 16 Studio MSK, Belluno, Italy; 17 Hospital Universitario Donostia, San Sebastian, Spain; 18 Dipartimento Di Diagnostica Per Immagini, Pineta Grande Hospital, Castel Volturno, Italy; 19 MSK Radiology Services, Heraklion, Crete Greece; 20 Hospital General Universitario de Valencia, Valencia, Spain; 21 2nd Department of Radiology, University General Hospital “ATTIKON” Medical School, National and Kapodistrian University of Athens, Haidari/Athens, Greece; 22 Dipartimento Di Scienze Biomediche Per La Salute, Università Degli Studi Di Milano, Milan, Italy; 23 Cambridge University Hospitals, Cambridge, UK; 24 Mid Cheshire Hospitals NHS Foundation Trust, Cheshire, UK; 25 Leeds Teaching Hospitals, Leeds, UK; 26 Department of Radiology, Special Hospital for Orthopedic Surgery and Traumatology St. Erazmo, Ohrid, North Macedonia; 27 Ss. Cyril and Methodius University in Skopje, Skopje, North Macedonia; 28 Imperial College Healthcare NHS Trust, London, UK; 29 Department of Radiology, Medical University Innsbruck, Innsbruck, Austria; 30 Oxford Musculoskeletal Radiology, Oxford University Hospitals, Oxford, UK; 31 Hospital Universitario Son Llatzer, Palma, Spain; 32 Hospital da Luz, Musculoskeletal Imaging Unit, Lisbon, Portugal; 33 AIRC, Advanced imaging research consortium, Lisbon, Portugal; 34 Division of Interventional Radiology, Istituto Europeo Di Oncologia, Milan, Italy; 35 Oxford Musculoskeletal Radiology, Oxford, UK; 36 Buckinghamshire Healthcare NHS Trust, Aylesbury, UK; 37 Department of Radiology, Charité-Universitätsmedizin Berlin, Berlin, Germany; 38 Department of Radiology, Herlev og Gentofte Hospital, Herlev, Denmark; 39 Hospital de Loulé, Loulé, Portugal; 40 Department of Oral Surgery, Wroclaw Medical University, Wroclaw, Poland; 41 Department of Radiology, Ospedale Evangelico Internazionale, Genoa, Italy; 42 Hospital POVISA, Vigo, Spain; 43 Post Graduate Institute of Medical Education & Research (PGIMER), Chandigarh, India; 44 Indywidualna Praktyka Lekarska Magdalena Posadzy, Poznan, Poland; 45 Department of Radiology, Lithuanian University of Health Sciences, Kaunas, Lithuania; 46 Institute of Radiology, University Medical Centre Ljubljana, Zaloska 7, 1000 Ljubljana, Slovenia; 47 Faculty of Medicine, University of Ljubljana, Ljubljana, Slovenia; 48 Department of Health Sciences, University of Genova, Genoa, Italy; 49 IRCCS Ospedale Policlinico San Martino, Genova, Italy; 50 Department of Radiology, AUVA Trauma Center Vienna, Vienna, Austria; 51 Radiology Dpt. MSK Unit. Hospital Clinic (CDIC), University of Barcelona (UB), Barcelona, Spain; 52 Clinical Center “Mother Theresa”, University Institute of Radiology, Skopje, North Macedonia; 53 Faculty of Medicine, Ss. Cyril and Methodius University in Skopje, Skopje, North Macedonia; 54 Radiology Department, Hospital ICOT Ciudad de Telde, Las Palmas, Spain; 55 Department of Medicine and Health Sciences, University of Molise, Campobasso, Italy; 56 Varelli Institute, Naples, Italy; 57 Guy’s and St Thomas’ Hospitals, London, UK

**Keywords:** Interventional radiology, Ultrasonography, Nerves, Lidocaine, Corticosteroid

## Abstract

**Objectives:**

To perform a Delphi-based consensus on published evidence on image-guided interventional procedures for peripheral nerves of the lower limb (excluding Morton’s neuroma) and provide clinical indications.

**Methods:**

We report the results of a Delphi-based consensus of 53 experts from the European Society of Musculoskeletal Radiology who reviewed the published literature for evidence on image-guided interventional procedures offered around peripheral nerves in the lower limb (excluding Morton’s neuroma) to derive their clinical indications. Experts drafted a list of statements and graded them according to the Oxford Centre for evidence-based medicine levels of evidence. Consensus was considered strong when > 95% of experts agreed with the statement or broad when > 80% but < 95% agreed. The results of the Delphi-based consensus were used to write the paper.

**Results:**

Nine statements on image-guided interventional procedures for peripheral nerves of the lower limb have been drafted. All of them received strong consensus. Image-guided pudendal nerve block is safe, effective, and well tolerated with few complications. US-guided perisciatic injection of anesthetic provides good symptom relief in patients with piriformis syndrome; however, the addition of corticosteroids to local anesthetics still has an unclear role. US-guided lateral femoral cutaneous nerve block can be used to provide effective post-operative regional analgesia.

**Conclusion:**

Despite the promising results reported by published papers on image-guided interventional procedures for peripheral nerves of the lower limb, there is still a lack of evidence on the efficacy of most procedures.

**Key Points:**

*• Image-guided pudendal nerve block is safe, effective, and well tolerated with few complications*.

*• US-guided perisciatic injection of anesthetic provides good symptom relief in patients with piriformis syndrome; however, the addition of corticosteroids to local anesthetics still has an unclear role*.

*• US-guided lateral femoral cutaneous nerve block can be used to provide effective post-operative regional analgesia. The volume of local anesthetic affects the size of the blocked sensory area*.

## Introduction

Over the last few years, increased interest in imaging the peripheral nerves has been supported by novel and high-performing ultrasound (US) transducers. High- and ultra-high frequency transducers produce high-resolution images, allowing the detection and thorough investigation of pathologic conditions of the peripheral nerves that once could only be evaluated with clinical examination and electroneurography tests [1–4]. Advances in US technology have also opened new frontiers in the management of neural disorders, given that image-guided percutaneous procedures may be considered in some neuropathies that are less suitable for surgical treatment [5, 6]. Specifically, US guidance is the preferred technique for perineural interventional procedures, which mostly consist of peripheral nerve blocks and interventions for entrapment neuropathies [5]. Indeed, despite the fact that unguided perineural interventions can be effective, there is a non-negligible risk of nerve injury or delivery of the medication too far away to have complete efficacy. Currently, several image-guided interventional procedures are routinely performed in clinical practice ranging from perineural corticosteroid injections for entrapment neuropathies to hydrodissection, aspiration of ganglia, phenol ablation, injection of botulinum toxin A, or alcohol blocks, and other minimally invasive procedures. Especially, US-guided treatments of peripheral entrapment neuropathies are rapidly emerging as an alternative option to surgery giving the possibility of symptoms relief comparable to a surgical release. Nevertheless, there is sparse evidence regarding the clinical value of these procedures and no clear guidelines have been produced to standardize how, when, and why image-guided interventions might be used for treating peripheral neuropathies of the lower limb. As already done for interventional procedures on the nerves of the upper limb [7], an expert board of the European Society of Musculoskeletal Radiology (ESSR) reviewed the evidence in the existing literature to compile evidence-based statements on clinical indications of image-guided procedures of the peripheral nerves of the lower extremities.

## Materials and methods

Institutional Review Board approval was not required as no patient data was used for this study. This paper is part of a larger project established by the Ultrasound and the Interventional Subcommittees of the ESSR to assess the published evidence on image-guided musculoskeletal interventional procedures in the lower limb and to produce a list of clinical indications [8]. An expert panel, selected from the members of these subcommittees, evaluated the existing literature as previously done in other ESSR consensus papers [7, 9–11]. They used a Delphi method of review, which consisted of rounds of literature evaluations by a panel of experts to state a list of agreed indications on a specific topic [12]. The AGREE II tool was used to ensure the quality of the Delphi method [13], which included the following steps:

1. Expert selection

The expert panel consisted of 53 radiologists from 16 countries (Austria, Belgium, Denmark, Germany, Greece, India, Italy, Lithuania, New Zealand, North Macedonia, Poland, Portugal, Slovenia, Spain, The Netherlands, UK), with established experience (from 5 to 35 years of experience in research activity) in musculoskeletal interventional procedures and in the scientific evaluation of medical literature. All experts were chosen from members of the Ultrasound and Interventional Subcommittees of the ESSR and divided into groups with a specific topic assigned to each group.

2. Literature search, statement drafting, and level of evidence

The literature search was performed on the major online databases (MEDLINE, Web of Science, EMBASE, and Google) including papers published up to the end of 2020, by using all the search terms relevant to the specific topic assigned to each group. The experts could also add papers found by screening the references of retrieved articles if considered relevant for their search. After the search, each group listed the evidence-based statements on their specific topic using the criteria of the Oxford Center of Evidence-Based Medicine in 2011 to identify the correct level of evidence for each statement [14]. The level of evidence may be graded down on the basis of study quality or graded up if there is large effect size, but, as a general rule, the level of evidence is generally identified as follows:

- Level 1: Systematic review of randomized trials
- Level 2: Prospective randomized trials
- Level 3: Non-randomized controlled cohort or follow-up study
- Level 4: Case-series, case–control studies, or historically controlled studies
- Level 5: Mechanism-based reasoning

3. Questionnaire preparation and consensus process

The drafted statements were revised by the coordinator of this project who disseminated a tool (Google Forms, Google LLC) to all experts sending a link via email. All experts accessed this tool through the link to agree, disagree, or abstain with the drafted statements. They were also invited to add any comments about the level of evidence and content of each statement. All answers were collected in an electronic spreadsheet (Microsoft Excel, Microsoft) and revised by the coordinator who modified the statements according to the experts’ comments. This was followed by a second round of discussion, with the same technique as the first. Any conflicts concerning the level of evidence and/or content of the statements that persisted after the second round of discussion were discussed via targeted emails sent to the involved experts and subsequently solved or kept.

*Data analysis and paper drafting*

After the rounds of discussion, the statements were once again shared with the experts to obtain consensus, which was considered strong when more than 95% of experts agreed with the statement or broad when more than 80% but less than 95% agreed [15]. The results of the Delphi-based consensus were used to write the paper that was shared with all panel members for final approval.

## Results

1. **Image-guided pudendal nerve block is safe, effective, and well tolerated with few complications.**

Level of evidence: 2

Agree, *n* = 53; disagree, *n* = 0; abstain, *n* = 0. Agreement = 100%

A successful pudendal nerve block is crucial for the diagnosis of pudendal neuralgia and provides guidance for treatment. Different studies, including two randomized controlled trials, demonstrated the absence of complications for this procedure [5, 16–21]. Furthermore, no significant difference in terms of efficacy was demonstrated between US and fluoroscopic guidance.

2. **US-guided interventional procedures around the genitofemoral nerve are feasible and effective, but evidence is only supported by a small series.**

Level of evidence: 4

Agree, *n* = 53; disagree, *n* = 0; abstain, *n* = 0. Agreement = 100%

US-guided procedures around the genitofemoral nerve are usually performed in conjunction with other nerve procedures (e.g. ilioinguinal, iliohypogastric nerves) [5, 22–28]; thus, in some papers, it is not easy to differentiate the outcomes of the various procedures. Furthermore, the results are reported on small series. Lee et al. reported the results of radiofrequency ablation of the genitofemoral nerve in four patients, with a reported 100% reduction of pain up to 12 months [24].

3. **US-guided perisciatic injection of anesthetic provides good symptom relief in patients with piriformis syndrome; however, the addition of corticosteroids to local anesthetics still has an unclear role.**

Level of evidence: 2

Agree, *n* = 53; disagree, *n* = 0; abstain, *n* = 0. Agreement = 100%

Several studies in patients with piriformis syndrome showed that US-guided perisciatic injection of anesthetic-corticosteroids is a safe procedure that provides good symptom relief [29–32]. In up to 50% of patients, symptoms may relapse, and additional perisciatic injections are warranted [30, 31, 33]. A prospective randomized controlled study in patients with piriformis syndrome showed that the addition of corticosteroid to anesthetic does not provide additional benefit [34]. On the other hand, a recent uncontrolled study on 30 patients showed that US-guided piriformis injection of corticosteroid-anesthetic seems to be effective for both somatic and neuropathic pain in piriformis syndrome patients at 1 week and 1 month [29].

4. **US-guided injection of botulinum toxin A in the piriformis muscle might offer long-term management of piriformis syndrome but evidence is limited; thus, its application in daily practice is still questionable.**

Level of evidence: 4

Agree, *n* = 53; disagree, *n* = 0; abstain, *n* = 0. Agreement = 100%

In the small case series by Rodríguez-Piñero et al., 6 months after the injection three patients were asymptomatic and three were feeling better [35]. Multiple injections may be administered over the course of treatment [36]. It has been shown that symptom relief after US-guided injections of botulinum toxin A into the piriformis muscle may be due to atrophy and fatty infiltration of the muscle [36].

5. **US-guided lateral femoral cutaneous nerve block can be used to provide effective post-operative regional analgesia. The volume of local anesthetic affects the size of the blocked sensory area.**

Level of evidence: 2

Agree, *n* = 53; disagree, *n* = 0; abstain, *n* = 0. Agreement = 100%

In a randomized trial, Nielsen et al. showed that US-guided lateral femoral cutaneous nerve block is safe and easy to perform with high success of analgesia [37]. Randomized controlled studies have shown improved postoperative experience in patients undergoing reconstructive surgery with lateral/anterolateral skin graft or as postoperative pain management in patients undergoing hip arthroplasty [38, 39]. However, in total hip arthroplasty, there is variability in anatomical placement of incision lines among different surgeons and it must be noted that there is limited analgesic effect if a posterior incision line is made [40]. A randomized study on healthy volunteers showed that the blocked sensory area is significantly larger when lateral femoral cutaneous nerve block is performed with 16 mL ropivacaine 0.75% than when it is performed with 8 mL ropivacaine 0.75% [41]. However, it does not result in greater coverage of the posterior or lateral incision lines used for total hip arthroplasty [41].

6. **US-guided corticosteroid perineural injections of the lateral femoral cutaneous nerve are an effective treatment option in the management of meralgia paresthetica.**

Level of evidence: 4

Agree, *n* = 53; disagree, *n* = 0; abstain, *n* = 0. Agreement = 100%

Two studies, both presenting a case series of 20 patients, showed that US-guided corticosteroid perineural injection is a good treatment option in the management of meralgia paresthetica. Tagliafico et al. showed resolution of symptoms in all patients 2 months after injection [42]; however, Klauser et al. showed complete resolution of symptoms in 75% of patients and partial resolution in 25% of patients at 12-month follow-up [43]. In the study by Klauser et al., a subgroup of patients with meralgia paresthetica underwent multiple sessions of lateral femoral cutaneous nerve corticosteroid injections at different levels (at anterior superior iliac spine, distal inguinal ligament, or lower thigh). The study showed that corticosteroid injections at multiple levels along the lateral femoral cutaneous nerve lead to a significantly better outcome at 12-month follow-up [43].

7. **Lateral femoral cutaneous nerve ablation may be effective in patients with intractable meralgia paresthetica and in patients after skin grafting from the lateral thigh, but further data is needed.**

Level of evidence: 4

Agree, *n* = 53; disagree, *n* = 0; abstain, *n* = 0. Agreement = 100%

Ethanol neurolysis, cryoneurolysis, and pulsed radiofrequency ablation are described in the literature as treatment options in patients with intractable meralgia paresthetica or in patients after skin grafting from the lateral thigh [44–47]. A recent retrospective review of six cases showed a substantial decrease in pain at 1 month (in some cases at 12 months) after US-guided radiofrequency ablation in patients with meralgia paresthetica resistant to conservative management [48]. The evidence is limited due to papers reporting small case series or case reports. No matter the method of ablation used, the papers report prolonged pain relief at follow-up.

8. **US-guided perineural injection of the first branch of the lateral plantar nerve (Baxter nerve) is a feasible procedure.**

Level of evidence: 4.

Agree, *n* = 52; disagree, *n* = 1; abstain, *n* = 0. Agreement = 98%

US-guided injection of the Baxter nerve was feasible in a cadaveric study and it could be considered for diagnostic and therapeutic purposes. No evidence of vascular injury was reported with the posterior-to-anterior approach [49]. During US-guided perineural injection of the Baxter nerve, the lateral plantar (82%), the medial calcaneal (17%), and the medial plantar nerves (8%) can be involved. This could make the procedure unspecific for diagnostic purposes [49].

9. **US-guided infrapatellar nerve block is a feasible, quick, and safe procedure to be used for post-arthroscopy analgesia. US-guided infrapatellar nerve hydro-dissection followed by corticosteroid injection could be useful in patients with persistent medial knee pain after total knee arthroplasty.**

Level of evidence: 4

Agree, *n* = 53; disagree, *n* = 0; abstain, *n* = 0. Agreement = 100%

The adjunct use of an infrapatellar nerve block is associated with improved pain relief after knee arthroscopy [50, 51]. No significant adverse effect or disadvantage was reported [50, 51]. The injection is performed at the level of its origin from the saphenous nerve [52, 53]. In combination with other nerve blocks, this procedure has proven to be useful as a pre-operative procedure for patellar fracture surgery [54].

Infrapatellar nerve injury is reported in 55–100% of patients with anterior knee paresthesia following total knee arthroplasty [55, 56]. Nerve hydro-dissection followed by corticosteroid injection may be a treatment option in patients with persistent pain after total knee arthroplasty. Pain relief remains consistent from one month to midterm follow-up; however, long-term outcomes of the treatment are unknown [56, 57].

## Discussion

Following a Delphi-based consensus, nine evidence-based statements on clinical indications of image-guided musculoskeletal interventional procedures of peripheral nerves of the lower limb were produced by an expert panel of the ESSR. None of the statements reached the highest level of evidence. Statements regarding image-guided interventional procedures to treat Morton’s neuroma have been reported in a separate paper [58].

This consensus paper on the published evidence regarding image-guided procedures of the peripheral nerves of the lower limb offers some interesting insights. Most published papers on this topic concern interventional procedures on the sciatic nerve and lateral femoral cutaneous nerve; for these nerves, prospective randomized clinical trials have shown that US-guided perineural injection of anesthetics provides good pain improvement in piriformis syndrome (statement #3) and effective post-operative analgesia (statement #5), respectively. Several image-guided interventional techniques have been described to treat the piriformis syndrome involving different targets, drugs, and volumes. Most authors agree that US-guided perisciatic injection of local anesthetics alone or combined with corticosteroids is a reasonable and effective option that can be considered as a therapeutic alternative before surgery, especially when the diagnosis is not fully clear, but also as a diagnostic procedure. Turning to the US-guided block of the lateral femoral cutaneus nerve, several authors have investigated the potential application of this procedure reporting its safety and effectiveness for reaching post-operative analgesia after reconstructive surgery with lateral/anterolateral skin graft or hip arthroplasty. This intervention may be considered for optimal comfort throughout the post-operative period improving the management of patients subjected to surgery in the hip/thigh region. However, only small retrospective studies have investigated the efficacy of alternative procedures on these two nerves. Specifically, initial promising results have been obtained by injecting botulinum toxin A under US guidance in patients with piriformis syndrome (statement #4), but this procedure needs to be supported by further data. Also concerning US-guided corticosteroid perineural injections (statement #6) and ablation (statement #7) of the lateral femoral cutaneous nerve in meralgia paresthetica, only a small series have highlighted the effectiveness of these procedures.

Regarding the other perineural procedures of the lower limb, only one statement on the efficacy and safety of image-guided pudendal nerve block reached level of evidence 2 (statement #1). Image-guided pudendal nerve block can be performed under either US or fluoroscopy guidance resulting in a safe and efficacious procedure. It can be used as a diagnostic test in patients with the suspect of pudendal neuralgia to treat pudendal nerve entrapment syndrome and to obtain effective analgesia before in different surgical settings, including pediatric circumcision, perineal surgery, and labour. All the other statements on the genitofemoral (statement #2), Baxter (statement #8), and infrapatellar (statement #9) nerves were supported only by small retrospective series. This confirms the need for further investigations to validate image-guided perineural procedures in clinical practice, similar to what is reported for the peripheral nerves of the upper limb [7].

Some limitations of this paper should be considered. First, this is not a detailed and thorough meta-analysis, the study design is an expert opinion that led us to draft a consensus document. We used a Delphi-based method of review of the existing literature for gathering experts’ consensus and establishing what is needed to do in the future to increase evidence on this topic. Then, no meaningful statistical analysis of the data was done; it was not required given that our results are based on statements drafting after literature review, expert’ consensus, and grading of the level of evidence.

In conclusion, nine evidence-based statements on image-guided perineural procedures of the lower extremities have been produced by an expert panel of the ESSR. The majority of statements discuss US-guided procedures on the sciatic nerve and lateral femoral cutaneous nerve. Despite the promising results reported by published papers on this topic, the lack of evidence in the existing literature on the efficacy of most procedures highlights the importance of supporting future prospective studies to clarify the clinical role of image-guided interventions on the peripheral nerves in the lower extremity.

## Abbreviations

ESSR: European Society of Musculoskeletal Radiology
US: Ultrasound

## Declarations

### Guarantor

The scientific guarantor of this publication is Luca Maria Sconfienza MD PhD.

### Statistics and biometry

No complex statistical methods were necessary for this paper.

### Methodology

• Literature-based Delphi process

## References

[1] Albano D, Aringhieri G, Messina C, De Flaviis L, Sconfienza LM (2020). High-frequency and ultra-high frequency ultrasound: musculoskeletal imaging up to 70 MHz. Semin Musculoskelet Radiol.

[2] Chianca V, Di Pietto F, Zappia M, Albano D, Messina C, Sconfienza LM (2020). Musculoskeletal ultrasound in the emergency department. Semin Musculoskelet Radiol.

[3] Picasso R, Zaottini F, Pistoia F (2020). High-Resolution Ultrasound of Small Clinically Relevant Nerves Running across the Posterior Triangle of the Neck. Semin Musculoskelet Radiol.

[4] Snoj Ž, Serša I, Maticic U, Cvetko E, Omejec G (2020). Nerve fascicle depiction at MR microscopy and high-frequency us with anatomic verification. Radiology.

[5] Tagliafico A, Bignotti B, Martinoli C (2016). Update on ultrasound-guided interventional procedures on peripheral nerves. Semin Musculoskelet Radiol.

[6] Silvestri E, Barile A, Albano D (2018). Interventional therapeutic procedures in the musculoskeletal system: an Italian Survey by the Italian College of Musculoskeletal Radiology. Radiol Medica.

[7] Sconfienza LM, Adriaensen M, Albano D (2020). Clinical indications for image guided interventional procedures in the musculoskeletal system: a Delphi-based consensus paper from the European Society of Musculoskeletal Radiology (ESSR)—part III, nerves of the upper limb. Eur Radiol.

[8] Sconfienza LM, Adriaensen M, Alcala-Galiano A (2021). Clinical indications for image guided interventional procedures in the musculoskeletal system: a Delphi-based consensus paper from the European Society of Musculoskeletal Radiology (ESSR) — part V, hip. Eur Radiol.

[9] Sconfienza LM, Albano D, Allen G (2018). Clinical indications for musculoskeletal ultrasound updated in 2017 by European Society of Musculoskeletal Radiology (ESSR) consensus. Eur Radiol.

[10] Sconfienza LM, Adriaensen M, Albano D (2020). Clinical indications for image-guided interventional procedures in the musculoskeletal system: a Delphi-based consensus paper from the European Society of Musculoskeletal Radiology (ESSR)—part I, shoulder. Eur Radiol.

[11] Sconfienza LM, Adriaensen M, Albano D (2020). Clinical indications for image-guided interventional procedures in the musculoskeletal system: a Delphi-based consensus paper from the European Society of Musculoskeletal Radiology (ESSR)—Part II, elbow and wrist. Eur Radiol.

[12] Steurer J (2011). The Delphi method: an efficient procedure to generate knowledge. Skeletal Radiol.

[13] Messina C, Vitale JA, Pedone L (2020). Critical appraisal of papers reporting recommendation on sarcopenia using the AGREE II tool: a EuroAIM initiative. Eur J Clin Nutr.

[14] Howick J, Chalmers I, Lind J, et al Oxford Centre for Evidence-Based Medicine 2011 Levels of Evidence.

[15] Sǎftoiu A, Gilja OH, Sidhu PS (2019). The EFSUMB guidelines and recommendations for the Clinical practice of elastography in non-hepatic applications: update 2018. Ultraschall der Medizin.

[16] Kale A, Usta T, Basol G, Cam I, Yavuz M, Aytuluk HG (2019). Comparison of ultrasound-guided transgluteal and finger-guided transvaginal pudendal nerve block techniques: which one is more effective?. Int Neurourol J.

[17] Wang X, Dong C, Beekoo D (2019). Dorsal penile nerve block via perineal approach, an alternative to a caudal block for pediatric circumcision: a randomized controlled trial. Biomed Res Int.

[18] Gaudet-Ferrand I, De La Arena P, Bringuier S (2018). Ultrasound-guided pudendal nerve block in children: a new technique of ultrasound-guided transperineal approach. Paediatr Anaesth.

[19] Bellingham GA, Bhatia A, Chan CW, Peng PW (2012). Randomized controlled trial comparing pudendal nerve block under ultrasound and fluoroscopic guidance. Reg Anesth Pain Med.

[20] Filler AG (2009). Diagnosis and treatment of pudendal nerve entrapment syndrome subtypes: Imaging, injections, and minimal access surgery. Neurosurg Focus.

[21] Xu J, Zhou R, Su W (2020). Ultrasound-guided bilateral pudendal nerve blocks of nulliparous women with epidural labour analgesia in the second stage of labour: a randomised, double-blind, controlled trial. BMJ Open.

[22] Huang Z, Xia W, Peng XH, Ke JY, Wang W (2019). Evaluation of ultrasound-guided genitofemoral nerve block combined with ilioinguinal/iliohypogastric nerve block during inguinal hernia repair in the elderly. Curr Med Sci.

[23] Kale A, Gurbuz Aytuluk H, Cam I, Basol G, Sunnetci B (2019). Selective spinal nerve block in ilioinguinal, iliohypogastric and genitofemoral neuralgia. Turk Neurosurg.

[24] Lee KS, Sin JM, Patil PP (2019). Ultrasound-guided microwave ablation for the management of inguinal neuralgia: a preliminary study with 1-year follow-up. J Vasc Interv Radiol.

[25] Frassanito L, Zanfini BA, Pitoni S, Germini P, Del Vicario M, Draisci G (2018). Ultrasound-guided genitofemoral nerve block for inguinal hernia repair in the male adult: a randomized controlled pilot study. Minerva Anestesiol.

[26] Ohgoshi Y, Takeda M, Miura M, Kori S, Matsukawa M (2017). Combination of femoral and genitofemoral nerve blocks is effective for endovascular aneurysm repair. J Clin Anesth.

[27] Campos NA, Chiles JH, Plunkett AR (2009) Ultrasound-guided cryoablation of genitofemoral nerve for chronic inguinal pain. Pain Physician 997–1000. 19935984

[28] Peng PWH, Tumber PS (2008) Ultrasound-guided interventional procedures for patients with chronic pelvic pain - a description of techniques and review of literature. Pain Physician 215–24. 18354713

[29] Terlemez R, Erçalik T (2019). Effect of piriformis injection on neuropathic pain. Agri.

[30] Rosales J, García N, Rafols C, Pérez M, Verdugo MA (2015). Perisciatic ultrasound-guided infiltration for treatment of deep gluteal syndrome: description of technique and preliminary results. J Ultrasound Med.

[31] Reus M, Dios Berná J, Vázquez V (2008). Piriformis syndrome: a simple technique for US-guided infiltration of the perisciatic nerve. Preliminary results. Eur Radiol.

[32] Jeong HS, Lee GY, Lee EG, Joe EG, Lee JW, Kang HS (2015). Long-Term assessment of clinical outcomes of ultrasound-Guided steroid injections in patients with piriformis syndrome. Ultrasonography.

[33] Walter WR, Burke CJ, Adler RS (2017). Ultrasound-guided therapeutic injections for neural pathology about the foot and ankle: a 4 year retrospective review. Skeletal Radiol.

[34] Misirlioglu TO, Akgun K, Palamar D, ErdenMG Erbilir T (2015). Piriformis syndrome: comparison of the effectiveness of local anesthetic and corticosteroid injections: a double-blinded, randomized controlled study. Pain Physician.

[35] Rodríguez-Piñero M, Vargas VV, Jiménez Sarmiento AS (2018). Long-term efficacy of ultrasound-guided injection of incobotulinumtoxin A in piriformis syndrome. Pain Med.

[36] Al-Al-Shaikh M, Michel F, Parratte B, Kastler B, Vidal C, Aubry S (2015). An MRI evaluation of changes in piriformis muscle morphology induced by botulinum toxin injections in the treatment of piriformis syndrome. Diagn Interv Imaging.

[37] Nielsen TD, Moriggl B, Barckman J (2018). The lateral femoral cutaneous nerve: description of the sensory territory and a novel ultrasound-guided nerve block technique. Reg Anesth Pain Med.

[38] Shank ES, Martyn JA, Donelan MB, Perrone A, Firth PG, Driscoll DN (2016). Ultrasound-guided regional anesthesia for pediatric burn reconstructive surgery: a prospective study. J Burn Care Res.

[39] Thybo KH, Mathiesen O, Dahl JB, Schmidt H, Hägi-Pedersen D (2016). Lateral femoral cutaneous nerve block after total hip arthroplasty: a randomised trial. Acta Anaesthesiol Scand.

[40] Nersesjan M, Hägi-Pedersen D, Andersen JH (2018). Sensory distribution of the lateral femoral cutaneous nerve block – a randomised, blinded trial. Acta Anaesthesiol Scand.

[41] Vilhelmsen F, Nersesjan M, Andersen JH (2019). Lateral femoral cutaneous nerve block with different volumes of Ropivacaine: a randomized trial in healthy volunteers. BMC Anesthesiol.

[42] Tagliafico A, Serafini G, Lacelli F, Perrone N, Valsania V, Martinoli C (2011). Ultrasound-guided treatment of meralgia paresthetica (lateral femoral cutaneous neuropathy): technical description and results of treatment in 20 consecutive patients. J Ultrasound Med.

[43] Klauser AS, Abd Ellah MMH, Halpern EJ (2016). Meralgia paraesthetica: ultrasound-guided injection at multiple levels with 12-month follow-up. Eur Radiol.

[44] Finneran JJ, Swisher MW, Gabriel RA (2020). Ultrasound-guided lateral femoral cutaneous nerve cryoneurolysis for analgesia in patients with burns. J Burn Care Res.

[45] Ahmed A, Arora D, Kochhar AK (2016). Ultrasound-guided alcohol neurolysis of lateral femoral cutaneous nerve for intractable meralgia paresthetica: a case series. Br J Pain.

[46] Chen CK, Phui VE, Saman MA (2012). Alcohol neurolysis of lateral femoral cutaneous nerve for recurrent meralgia paresthetica. Agri.

[47] Fowler IM, Tucker AA, Mendez RJ (2012). Treatment of meralgia paresthetica with ultrasound-guided pulsed radiofrequency ablation of the lateral femoral cutaneous nerve. Pain Pract.

[48] Abd-Elsayed A, Gyorfi MJ, Ha SP (2020). Lateral femoral cutaneous nerve radiofrequency ablation for long-term control of refractory meralgia paresthetica. Pain Med.

[49] Presley JC, Maida E, Pawlina W, Murthy N, Ryssman DB, Smith J (2013). Sonographic visualization of the first branch of the lateral plantar nerve (Baxter nerve) technique and validation using perineural injections in a cadaveric model. J Ultrasound Med.

[50] Lundblad M, Forssblad M, Eksborg S, Lönnqvist PA (2011). Ultrasound-guided infrapatellar nerve block for anterior cruciate ligament repair: a prospective, randomised, double-blind, placebo-controlled clinical trial. Eur J Anaesthesiol.

[51] Hsu LP, Oh S, Nuber GW (2013). Nerve block of the infrapatellar branch of the saphenous nerve in knee arthroscopy a prospective, double-blinded, randomized, placebo-controlled trial. J Bone Joint Surg Am.

[52] Lundblad M, Kapral S, Marhofer P, Lönnqvist PA (2006). Ultrasound-guided infrapatellar nerve block in human volunteers: description of a novel technique. Br J Anaesth.

[53] Gong W, Wang A, Fan K (2019). A simple and novel ultrasound-guided approach for infrapatellar branch of the saphenous nerve block. J Clin Anesth.

[54] Kim Y-M, Kang C, Joo Y-B, Yeon K-U, Kang D-H, Park I-Y (2015). Usefulness of ultrasound-guided lower extremity nerve blockade in surgery for patellar fracture. Knee Surg Relat Res.

[55] Sundaram RO, Ramakrishnan M, Harvey RA, Parkinson RW (2007). Comparison of scars and resulting hypoaesthesia between the medial parapatellar and midline skin incisions in total knee arthroplasty. Knee.

[56] Shi SM, Meister DW, Graner KC, Ninomiya JT (2017). Selective denervation for persistent knee pain after total knee arthroplasty: a report of 50 cases. J Arthroplasty.

[57] Clendenen S, Greengrass R, Whalen J, O’Connor MI (2015). Infrapatellar saphenous neuralgia after TKA can be improved with ultrasound-guided local treatments. Clin Orthop Relat Res.

[58] Sconfienza LM, Adriaensen M, Albano D (2021). Clinical indications for image guided interventional procedures in the musculoskeletal system: a Delphi-based consensus paper from the European Society of Musculoskeletal Radiology (ESSR) — part VI, fott and ankle. Eur Radiol.

